# Computational Model for Analysing the Tooth Deflection of Polymer Gears

**DOI:** 10.3390/polym16050677

**Published:** 2024-03-02

**Authors:** Aljaž Ignatijev, Srečko Glodež, Janez Kramberger

**Affiliations:** Faculty of Mechanical Engineering, University of Maribor, Smetanova 17, 2000 Maribor, Slovenia; aljaz.ignatijev@student.um.si (A.I.); janez.kramberger@um.si (J.K.)

**Keywords:** polymer gears, tooth deflection, computational modelling

## Abstract

A computational model for analysing the tooth deflection of polymer gears is presented in this paper. Because polymer gears have less stiffness compared to metal gears, the proposed approach considers a comprehensive analysis to determine the most suitable numerical model, i.e., the number of teeth that should be modelled for a given gear’s geometry and material. The developed computational model has been evaluated using a spur gear pair, where the pinion made of POM was meshed with a support gear made of steel. Material properties were assigned with linear elastic characteristics for the gear, while the pinion was characterised by hyperelastic properties using POM material. Furthermore, a nonlubricated frictional contact between the gear and pinion was considered in the numerical computations. The computational results that were obtained were compared to the empirical results according to VDI 2736 guidelines. Here, the computational approach showed more accurate results due to the hyperelastic material characteristics of POM and the simulation of multiple teeth meshing. However, VDI 2736 calculation showed comparability with the computational results, with a slightly larger deviation at higher loads. In this respect, the proposed computational approach is more suitable for analysing the tooth deflection of polymer gears under higher loads.

## 1. Introduction

Polymer gears are often used in different engineering applications, such as E-bikes, linear actuators, industrial and robotics automation, computer components, medical facilities, the food industry, etc. [1,2,3,4]. Polymer gears can be manufactured by cutting or injection moulding. The latter is typical for large series production. The main advantages of polymer gears include dramatic weight reduction and cost savings, low coefficient of friction, self-lubrication features, reduced noise, high resistance against impact loading, damping of vibration, ability to be used in food preparation areas, etc. However, polymer gears also have some disadvantages. The most important, if compared to metal gears, is their lower load-carrying capacity and low operating temperatures. Furthermore, moulded gears present some difficulties in achieving high tolerances, which are also dependent on temperature and humidity conditions [5,6,7,8,9].

**Figure 1:**
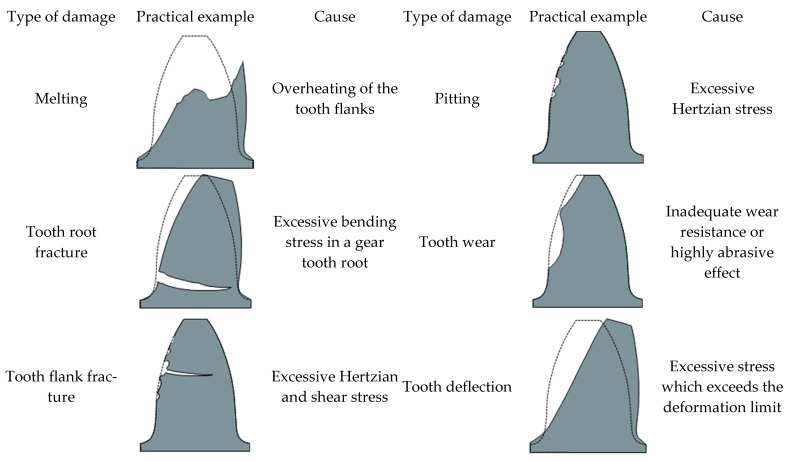
Typical failures of polymer gears and their causes according to VDI 2736-2.

The calculation procedure to obtain the load-carrying capacity of polymer gears is usually based on the standardised procedure according to the VDI 2736 guidelines [10,11,12,13]. It is recommended that a check calculation of the load-carrying capacity be made for the central position of the components within the tolerance field. Furthermore, the following failure types of polymer gears are addressed in [14,15,16] and explained additionally in [17,18,19]: melting, tooth root fracture, tooth flank fracture, pitting, tooth wear and tooth deflection (see Figure 1). However, the proposed research focuses mainly on the tooth deflection of polymer gears which is a crucial parameter in respect to the proper gear drive operation. Namely, excessive tooth tip deflection can lead to serious disturbances of gear meshing and, consequently, increased noise and wear on the teeth flanks [20,21,22].

Many researchers have recently investigated different types of polymer gears concerning tooth deflection using the appropriate computational approaches. Trobentar et al. [23] investigated the gear tooth deflection of spur polymer gears made of POM. Their study determines the tooth deflection behaviour using the Young’s elastic material model and the hyperelastic Marlow model. The computational analyses have shown that the appropriate FE model (corresponding number of analysed gear teeth) significantly influences the correct stiffness of the analysed gear pair. The computational results also indicated that the appropriate non-linear material model should be considered, especially in the case of higher contact forces and, consequently, large deflections of gear teeth. The same authors investigated the influence of surface coatings on the tooth tip deflection of polymer gears [24]. The obtained numerical results were then used to define approximate empirical equations for the calculation of gear tooth tip deflection for the coating material and the thickness of the surface coating layer. The results showed that the tooth tip deflection decreases with large values of the coating material (Young’s modulus) and with the coating layer thickness. Melick et al. [25] investigated the load sharing and stresses in steel–plastic gear pairs, revealing significant deviations from conventional steel gear theories, which led to results similar to those found in this study. Load sharing in steel–plastic pairs showed asymmetry around the pitch point due to tooth deformation, with the most severe loading occurring during the last part of the meshing cycle. According to these findings, Vignesh et al. [26,27] attempted to establish the highest load a polymer gear mechanism could endure without triggering contact extension resulting from deflection. Their objective was to lower wear and improve the process of selecting appropriate loads for polymer gear systems. The authors investigated the behaviour of extended contact, including its effects on kinematics, bending stiffness, stresses, and gear twist angle. They used finite element analysis for quasi-static analysis of two-dimensional teeth, introducing a new dimensionless parameter for predicting extended contact. Karimpour et al. [28] proposed a computational model to analyse the meshing behaviour of polymer gears using the Finite Element Method (FEM). The numerical simulations showed that the kinematic behaviour of polymer gears is significantly different from those predicted by the classical metal gear theory. Namely, extensions to the path of contact occur at the beginning and end of the meshing cycle, which is caused by large tooth deflections of polymer gear teeth due to much lower stiffness values compared to metallic gears.

Many authors have also investigated the tooth deflection of polymer gears based on experiments, focusing on the development of new experimental methods. Herzog et al. [29] tested cylindrical gears made of polybutylene terephthalate (PBT) under various loading torques using a newly developed experimental in situ system, which is capable of measuring tooth deformations. Their results showed that long-term gear tests under varying rotational speeds and loading lead to increasing wear and tooth deflection at higher speeds and torques. In further work [30], the same authors tested a gear pair of steel/PBT at different rotational speeds and temperatures, focusing on measuring elastic tooth deflections. The experimental results that were obtained were then compared with VDI 2736 and Dynamomechanical Analysis (DMA). It was found that the measured tooth deflection at lower speeds was significantly lower than the calculated deformation according to VDI 2736 guidelines. On the other hand, Črne et al. [31] used a gear pair of steel/POM-C in their research, comparing the DIC and EDD methods and verifying their adequacy using the Finite Element Method (FEM).

In the framework of the presented study, a computational model for analysing the tooth deflection of polymer gears was developed and evaluated using a spur gear pair, where the pinion made of POM was meshed with a support gear made of steel. The computational analysis was divided into two steps. Firstly, a transient simulation was performed to identify the Highest Point of Single Tooth Contact (HPSTC), which represents the most critical engagement point regarding tooth deflection. In the next step, the numerical simulation was performed to obtain the tooth tip deflection.

The latest research advances the analysis of tooth deflection in polymer gears using a sophisticated computer model. This study differs from traditional linear elastic models and includes a hyperelastic model fitted to the POM gear, which allows a more accurate representation of the material behaviour. Furthermore, this study addresses the limitations of conventional approaches, such as VDI 2736, which are noticeable, especially at lower loads, and improves the methodology for tooth deformation analysis. In some previous studies described above, the authors either neglected the hyperelastic material properties or did not consider the influence of multi-tooth meshing. Trobentar et al. [23] included a hyperelastic material model for polymer gears, but the influence of multiple teeth in the meshing has not been considered, which is particularly noticeable for smaller modules. Similarly, Vignesh et al. [27] focused on numerical simulations related to multi-tooth meshing but did not consider the influence of material properties, such as hyperelastic behaviour. In that respect, the proposed article explains the optimisation of the computational model, resulting in more accurate simulation results and reasonable computational times. The research highlights the importance of a computational approach, particularly when considering the hyperelastic properties of POM, thus introducing new insights into the analysis of polymer gears. Notably, the computational model highlights phenomena such as contact path extension and additional loads on neighbouring teeth, explaining how loads distribute across multiple teeth, an important aspect in polymer gear performance assessment.

## 2. Materials and Methods

### 2.1. Material and Geometry of Analysed Gear Pair

**Figure 2:**
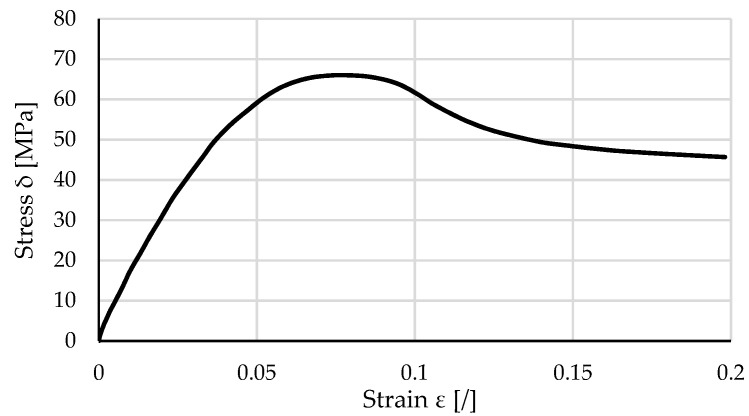
Stress–strain diagram of POM.

The developed computational model has been evaluated using a spur gear pair, where the pinion made of POM was meshed with a support gear made of steel [32]. The basic geometrical and material parameters of the analysed gear pair are presented in Table 1. The numerical simulation was based on a hyperelastic model considering the stress–strain diagram shown in Figure 2, taken from the VDI 2736-1 guideline [10].

### 2.2. Determination of Tooth Deflection According to VDI 2736 Guidelines

The standardised procedure VDI 2736-2 [11] applies to cylindrical polymer gears with reference profiles in accordance with DIN 867 [34] and DIN 58400 [35], as well as with modules *m*_n_ ≥ 0.1 mm. The calculation of load-carrying capacity is based on the Standard DIN 3990 [36], which basically applies to metallic materials. In comparison with metals, polymers have a number of special features, such as (i) the dependence of their mechanical properties on operating temperature as well as on the level, time, and speed of loading; (ii) their poorer thermal conductivity; and (iii) greater deformation. Considerable tooth deflection may occur, particularly with polymer gears with a narrow face width, such as those used in precision transmissions. This is due to their modulus of elasticity being much smaller than that of metals. It may take the form of pitch deviations, cause meshing impacts, and, among other things, increase noise. Furthermore, if the teeth of a polymer gear have already been exposed for a relatively long time to a stationary load, the permissible tooth root stress could be exceeded due to the creep. In this respect, the deflection of the tooth tip should satisfy the following condition [11]:

(1) $$\lambda =\frac{7.5\cdot F_{t}}{b\cdot cos\beta }\cdot \left(\frac{1}{E_{1}}+\frac{1}{E_{2}}\right)\;\leq \;\lambda_{P}$$

where *λ* is the tooth tip deflection, *λ_P_* is the permissible tooth tip deflection, *F*_t_ is the nominal tangential force, *b* is the face width, β is the helix angle at the reference circle, and *E*_1_ and *E*_2_ are the moduli of elasticity of the pinion (1) and gear (2), respectively. A guide value for the permissible tooth tip deflection is [11]:

(2) $$\lambda_{P}\approx 0.07\cdot m_{n}$$

where *m_n_* is the normal module. If condition (2) is not satisfied (*λ* > *λ_P_*), running noise may increase and service life be reduced. At greater levels of tooth deflection, tip retraction may moderate the negative effects arising from deflection.

### 2.3. Computational Modelling

#### 2.3.1. Geometry

**Figure 3:**
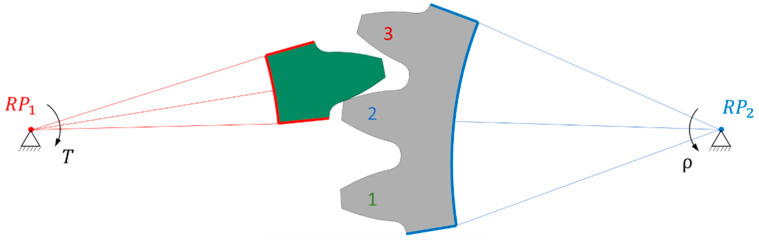
Computational 1/3 model of gear pair.

**Figure 4:**
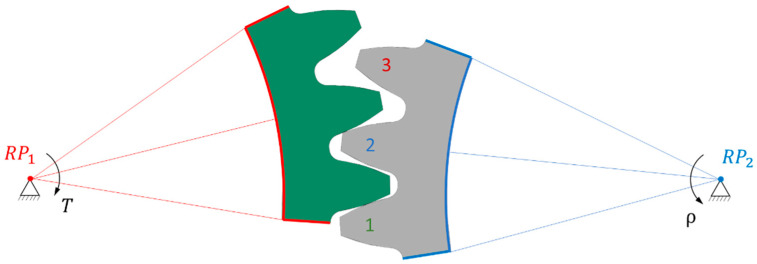
Computational 3/3 model of gear pair.

**Figure 5:**
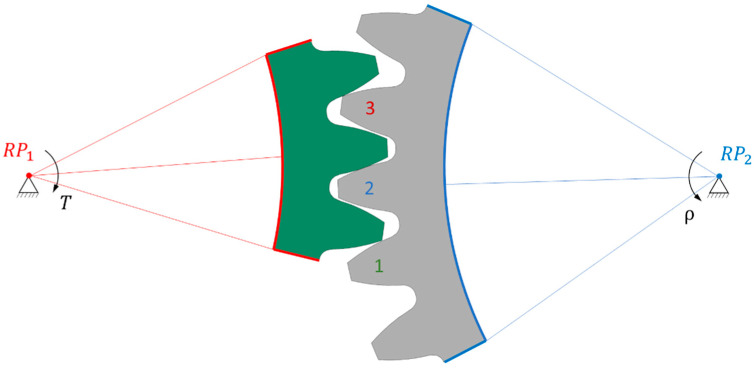
Computational 3/5 model of gear pair.

Because polymer gears have less stiffness compared to steel ones, we implemented a comprehensive analysis to determine the most suitable model. Our approach involved creating several 2D geometric models, each with its own characteristics. The first model (1/3) featured only one gear tooth and three pinion teeth (Figure 3); the second model (3/3) included three gear teeth and three pinion teeth (Figure 4), while the third model (3/5) comprised three gear teeth and five pinion teeth (Figure 5). In Figure 3, Figure 4 and Figure 5, the teeth referred to in the following text are identified by a sequential number.

#### 2.3.2. Boundary Conditions

The boundary conditions were defined in remote points placed in the axis of the gears. The trimmed sides of the gear, marked in red, were rigidly connected to remote point RP_1_, allowing only rotation around the gear axis and the addition of a torque *T* = 16 Nm. On the other side, the trimmed sides of the pinion, marked in blue, were rigidly connected to remote point RP_2_, where they were fixed in all directions of the coordinate system. It is important to note that a rigid connection was used for linking remote points RP_1_ and RP_2_, as a deformable connection would cause the gear to deviate from the gear axis, leading to inaccurate results.

Frictional contact between the gear and pinion was defined with a coefficient of friction μ = 0.2, as given in VDI 2736 for dry (nonlubricated) contact of gear flanks. To specifically analyse the normal force on the gear tooth, frictionless contact conditions were employed. Material properties were assigned with linear elastic characteristics for the gear, while the pinion was characterised by hyperelastic properties using POM material.

#### 2.3.3. Finite Element Mesh

**Figure 6:**
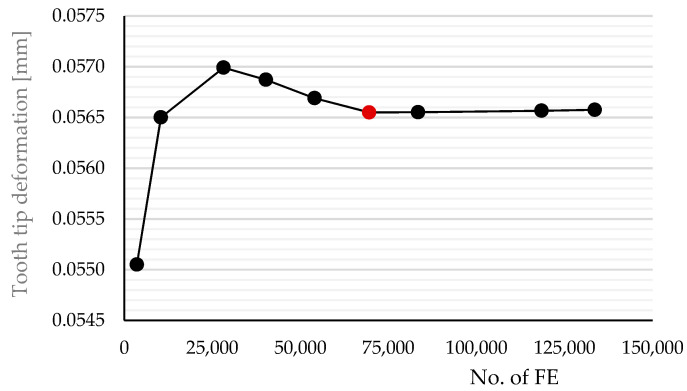
Finite element mesh convergence analysis of 3/5 model.

**Figure 7:**
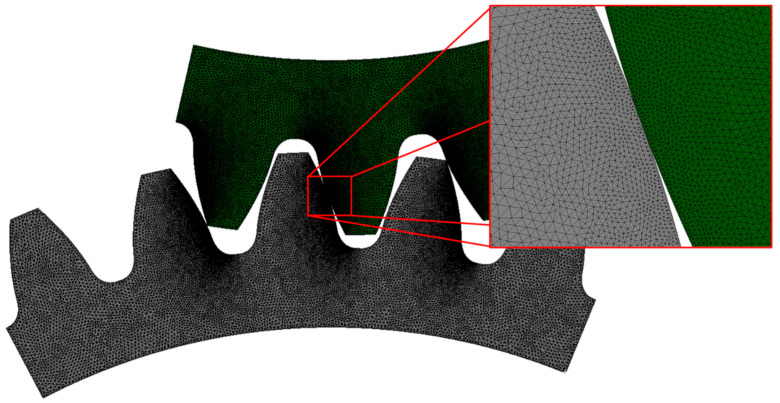
Finite element mesh of gear pair 3/5 model.

The FE mesh was generated using triangular linear finite elements. The mesh was denser in the contact area, while a larger finite element size was chosen in the rest of the gears. A convergence analysis (see Figure 6) helped determine the optimal global element size of 0.1 mm and a local element size of 0.02 mm. The mesh was refined with the local size on the edges of tooth contacts (see Figure 7 for 3/5 model). The finite element mesh contained 69,513 FE (red dot). A similar procedure has also been performed for models 1/3 and 3/3.

#### 2.3.4. Numerical Simulation

**Figure 8:**
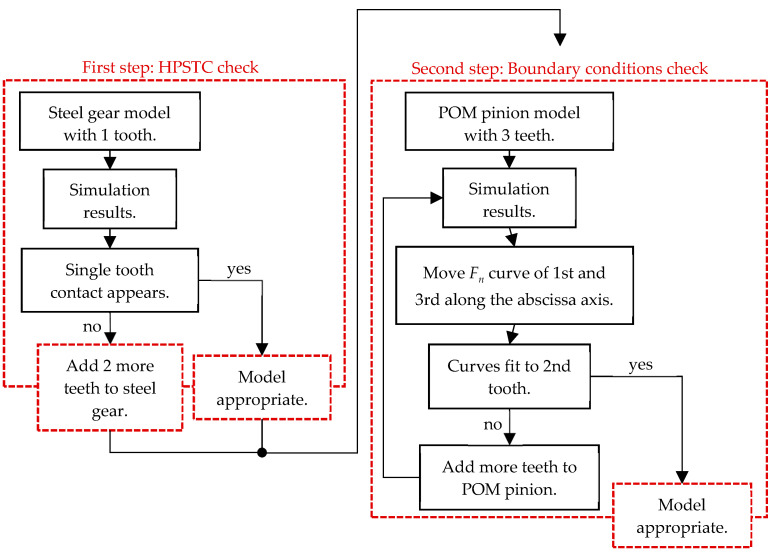
Flowchart of the computational model relevance check.

Due to the lower stiffness of polymer materials, a model relevance was checked (Figure 8). This includes verifying whether the Highest Point of Single Tooth Contact (HPSTC) occurs or not and checking the appropriateness of the model simplification. This is because, in polymer materials, loads are distributed over a larger area of the gear rather than just the meshing tooth [25].

The analysis of normal contact forces on the gear teeth was performed in two steps. Firstly, a transient simulation was conducted, placing the gear at the start of contact (tip) of the second pinion tooth, followed by a rotation around RP_2_ of 18°. Then, a static analysis was carried out, placing the gear in a position where the maximum force acted on the second pinion tooth, as shown in Figure 2, Figure 3 and Figure 4. The numerical simulation was performed using the software package ANSYS version 2023 R1 [37].

## 3. Results and Discussion

### 3.1. Model Comparison

**Figure 9:**
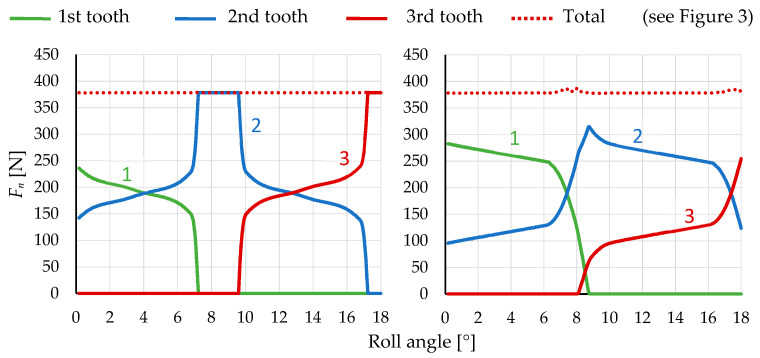
Normal force *F*_n_ on 3 neighbouring pinion teeth in dependence on roll angle of pinion of steel/steel (**left**) and steel/POM (**right**) meshing gear pair.

In the comparison between POM and a steel pinion, it is evident that a single gear tooth is insufficient. In the case of the POM pinion, the point of single tooth contact is never reached due to the deformation of neighbouring teeth over the pinion’s body, as opposed to what occurs in the case of a steel pinion (Figure 9). By applying the 3/3 and 3/5 models, it was observed that, at the maximum force acting on the second (blue curve) tooth, the first (green curve) and third (red curve) teeth still remain in contact the whole time. This occurrence is a result of the entire pinion body deforming in the direction of the normal force due to the lower stiffness of POM. 

**Figure 10:**
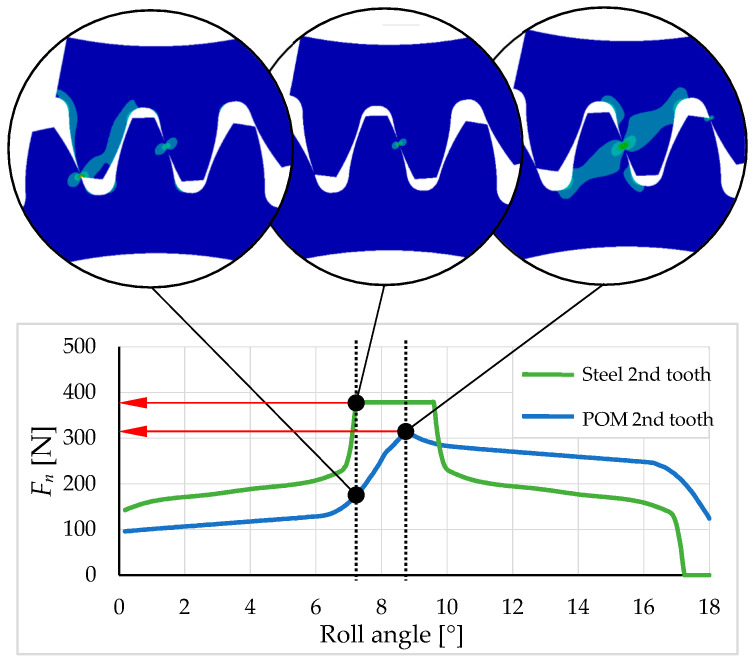
Normal force *F_n_* on 2nd pinion tooth in dependence on roll angle of pinion of steel/steel (green curve) and steel/POM (blue curve) meshing gear pair comparison.

Figure 10 shows the difference between a steel pinion and a POM pinion. Compared to steel, POM has a lower maximum normal force, and, consequently, its location on the tooth flank shifts closer to the root of the tooth. Similar findings were previously identified by [25].

It is evident from Figure 10 that the contact path has been extended due to the tooth deviation. This can be seen on the abscise axis, where contact is still present at a rolling angle of 18° relative to the POM tooth. However, according to the theory of steel gears, in this case, the contact separation already occurs at about 17° of rolling angle.

**Figure 11:**
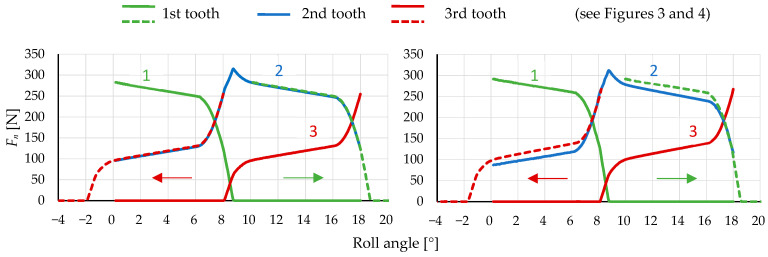
Normal force *F*_n_ on 3 neighbouring pinion teeth in dependence on the roll angle of 3/5 model (**left**) and 3/3 model (**right**) and shifted curves of teeth 1 and 3 along the abscissa axis.

During the analysis of model relevance, the results were observed by varying the normal force on the pinion teeth while attempting to overlay curves by shifting them along the abscissa axis (roll angle) on the diagram, as shown in Figure 11 with arrows. In the 3/3 model, it was noticeable that the curves of the first and third teeth were shifted higher than the curve of the second tooth, indicating a higher reaction force on the pinion tooth. This suggests the irrelevance of the model since the force on all gear teeth during engagement should follow a similar pattern. The cause of these differences lies in the fixed boundary condition, which is too close to the first and third teeth, preventing actual deformation around the tooth. Adding two additional pinion teeth to the model on each side enables the deformation of a larger area around the loaded tooth. Therefore, the decision was made to use the 3/5 model, and its results are shown in Figure 11 on the right, where the curves would perfectly fit if appropriately shifted along the abscissa axis, as shown in Figure 11 on the left. Adding additional teeth and the rest of the gears and pinion body resulted in negligible differences. The dotted curve in Figure 10 represents the total reaction force on the pinion. At around 7.5° and 8.5° of POM roll angle, a peak appears on the curve, which leads to transmission error due to a force transfer from one tooth to another.

### 3.2. Comparison between Numerical and Analytical (VDI 2736) Results

**Figure 12:**
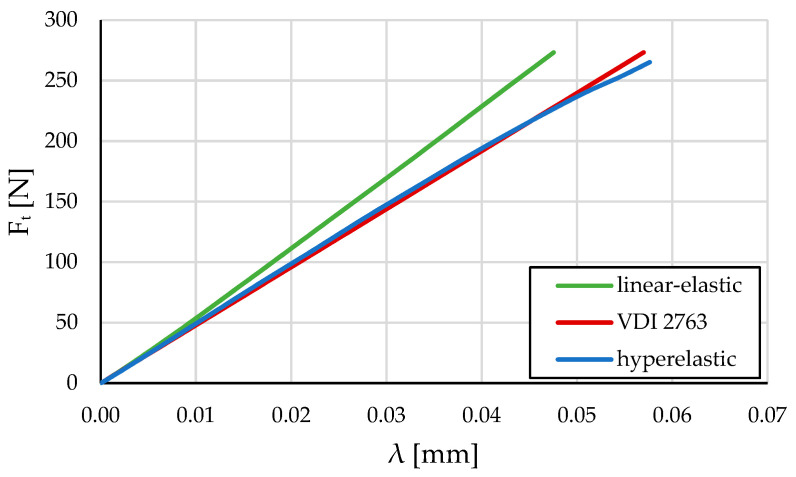
Tangential force on pinion tooth *F*_t_ at the highest point of maximal force in dependence on tooth deformation λ.

Figure 12 shows a comparison of the results from the 3/5 model with a linear-elastic material model and a hyperelastic material model, as well as calculations according to VDI 2736 guidelines. However, as previously determined, VDI 2736 does not provide accurate results. The hyperelastic curve of the 3/5 model fits well with the VDI 2736 curve, while the linear-elastic model 3/5 curve is considerably far from VDI 2736. According to these findings, it can be concluded that using a linear-elastic model would worsen the accuracy in comparison to the hyperelastic material model.

Based on the obtained computational results, it is clear that VDI 2736 serves as a good approximation for calculating the deformation of the polymer gear tooth but not along the entire engagement line and the magnitude of the external loading (i.e., torque). Due to the consideration of the hyperelastic model, the deviation of the curve increases at higher loads (at approx. 250 N and above).

According to Equation (2), the permissible tooth tip deflection values would be 0.175 mm. Since the hyperelasticity curve starts to deviate from the VDI 2736 calculation curve at higher loads, this calculation would give a much higher permissible tangent force for this value of permissible tooth tip deflection. It can be concluded that, given this choice of material and geometry, VDI 2736 is only applicable to lower loads.

The material parameters and the hyperelastic material model are derived from VDI 2736-1. Since VDI 2736 is based on material parameters determined previously from experiments, we can already conclude their adequacy. A numerical model with the same parameters has been used for comparability with the analytical results of VDI 2736. However, the fact that VDI 2736 gives a linear progression of the results allows us to conclude the limitations of VDI 2736. Since the numerical results give results comparable to the analytical calculation, we can conclude that the model is adequate.

**Figure 13:**
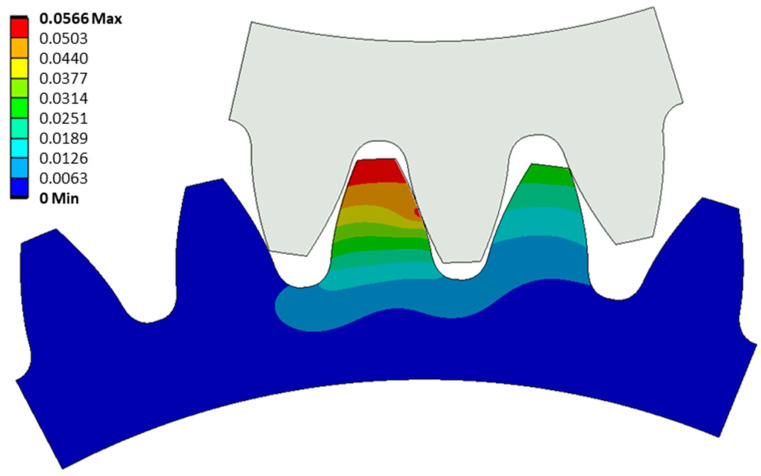
Deformation of pinion at the highest point of maximum force on 2nd tooth.

The maximum tooth deflection at the highest point of the tangential force on the second tooth occurs at the tip of the tooth and measures 0.057 mm (Figure 13). Through the static analysis, it was observed that the gear deforms the second and third pinion teeth, while the first tooth deviates from contact with the gear due to the deformation of a larger area of the pinion body.

## 4. Conclusions

The presented study proposes a computational model to analyse the tooth deflection of polymer gears. It was shown that an incorrectly defined numerical model for polymer gear pairs results in entirely inaccurate results, emphasising the need to carefully examine the dynamic behaviour of gears. In this case, the use of a model where only one gear tooth meshes with the pinion is irrelevant. Unlike stiffer materials, in the case of a pinion made of POM, a single contact point never occurs due to deformation with such geometry. Not only is the tooth deformed, but it is also part of the pinion body, which causes it to deform and “drag along” the neighbouring teeth. Consequently, a smaller normal contact force on a single tooth can be obtained, and the point of the highest maximal normal contact force moves closer to the tooth root, which leads to asymmetric tooth meshing and contact path extension.

The relevance of the numerical model can be determined by comparing the reaction forces on each individual tooth. Since the tooth meshing cycle occurs in a consistent cyclic pattern, the reaction forces should be equal on each tooth of the gear. Therefore, by aligning the curves of the reaction force with respect to the gear rotation angle in a properly designed numerical model, we achieve a perfect overlap. This implies that the reaction forces on the gear are equal at every point on all teeth. When choosing a material model, it is necessary to check the simplification to linear elasticity. In our case, using a linear-elastic material model would reduce the accuracy.

The main purpose of the work was to create a suitable computational model comparable to the calculation based on VDI 2736 guidelines. Here, numerical results showed more accurate results due to the hyperelastic material characteristics of POM. VDI 2736 calculation showed the comparability with numerical results with a slightly larger deviation at higher loads. Despite this, the VDI 2736 guidelines serve as an appropriate calculation approach to check the tooth deflection of polymer gears.

## Figures and Tables

**Table 1:** Basic parameters of the analysed gear pair.

Parameter	Tested Gear	Supported Gear
Material	POM	Steel (16MnCr5)
Normal module *m*	2.5 mm	2.5 mm
Pressure angle α_n_	20°	
Helix angle β	0°	
Number of teeth *z*	36	36
Tooth width *b*	14 mm	14 mm
Profile shift coefficient *x*	0	0
Centre distance *a*	90 mm	
Basic rack profile	ISO 53 [33]	
Young’s modulus *E*	2600 MPa	210,000 MPa
Poisson’s ratio ν	0.386	0.280

## Data Availability

Data are contained within the article.
